# Solid-State Yeast Fermented Wheat and Oat Bran as A Route for Delivery of Antioxidants

**DOI:** 10.3390/antiox8090372

**Published:** 2019-09-04

**Authors:** Lavinia Florina Călinoiu, Adriana-Florinela Cătoi, Dan Cristian Vodnar

**Affiliations:** 1 Faculty of Food Science and Technology, University of Agricultural Sciences and Veterinary Medicine Cluj-Napoca, Calea Mănăștur 3-5, 400372 Cluj-Napoca, Romania; 2 Institute of Life Sciences, University of Agricultural Sciences and Veterinary Medicine Cluj-Napoca, Calea Mănăștur 3-5, 400372 Cluj-Napoca, Romania; 3 Department of Pathophysiology, “Iuliu Haţieganu” University of Medicine and Pharmacy Cluj-Napoca, Victor Babeş street 3-4, 400012 Cluj-Napoca, Romania

**Keywords:** bioprocess, solid-state fermentation, yeast, wheat bran, oat bran, phenolic acids, enzymes, sustainability, low cost, antioxidants enrichment

## Abstract

The purpose of our study was to evaluate the potential of solid-state yeast fermentation (SSYF) in improving the phenolic acid content and composition, and the antioxidant activity of commercial wheat bran (WB) and oat bran (OB). The ultrasound-assisted methanolic extracts were compared for their total phenolic content (TPC), phenolics composition, and in vitro antioxidant activity in order to study the effect of fermentation time on the chemical profile and activity of bioactive compounds. The comparative analysis revealed significant differences (*p* < 0.05) between days of fermentation (0 through 6). The highest TPCs were obtained on day 3 for WB (0.84 ± 0.05 mg of gallic acid equivalents [GAE]/g dry weight [DW]), and on day 4 for OB (0.45 ± 0.02 mg GAE/g DW). The highest relative percentage increase in the phenolics concentration of WB was also registered on day 3 (ferulic acid +56.6%, vanillic acid +259.3%, dihydroxybenzoic acids +161.2%, apigenin-glucoside +15.3%); for OB, this was observed on day 4 (avenanthramide 2f +48.5%, ferulic acid +21.2%). Enhanced antioxidant activities were significantly correlated with the highest TPCs. Our results suggest that SSYF may be a useful procedure for enrichment of antioxidants in cereal bran, considering the design of different functional foods and nutraceuticals.

## 1. Introduction

Wheat (*Triticum aestivum* L. and *Triticum durum* L.) is a highly cultivated crop (>218 million ha), being the most consumed staple food with a worldwide annual production of >717 million tons in 2017. Oat (*Avena sativa* L.), even if consumed in lower quantities, has an increasing production trend (>25 million tons in 2017) [1] due to its recognition as a healthy food with high bioactive content [2]. Wheat and oat whole grains, and specifically their outer parts, are considered rich sources of phenolic compounds with significant antioxidant activity [3,4].

Plant-based natural antioxidants may act as replacers for synthetic antioxidants, being able to enhance the natural resistance of the body against reactive oxygen species (ROS) damage [5]. There have been several meta-analyses of epidemiological data suggesting that consumption of phenolic-rich food in humans may reduce the risk of non-communicable diseases, such as diabetes, cardiovascular disease, obesity, and some cancers [6,7,8,9]. Antioxidants can increase protection against these diseases in several ways: quenching free radicals, chelating transition metals, stimulating antioxidative enzymes, and reducing peroxides. The fibers present in whole grains act as synergistic components, also contributing to disease risk reduction [10].

In the cereal-processing industry, the wastage rate is at 30% (286 million tons) [1], mainly represented by bran tissues. Recent advances in biotechnology ensure that food processing waste is perceived as a rich source of bioactive compounds [11,12,13,14,15,16,17,18]. The major phenolic compounds found in wheat and oat are concentrated in the bran parts and belong to phenolic acids and flavonoids sub-classes. Phenolic acids are represented in high percentages by ferulic acid, caffeic acid, vanillic acid, *p*-coumaric acid, sinapic acid, dihydroxybenzoic acid, and avenanthramide (2c, 2p, 2f) [3,19]. Oat is the only natural source of water-soluble avenanthramides (alkaloids containing phenol groups) with very strong antioxidant capacity in vitro and in vivo [20]. In both matrices, ferulic acid can reach up to 46–94.6% of the total polyphenols, being influenced by variety, geographical region, crop conditions, and extraction methods [21]. Wheat bran (WB) and oat bran (OB) are among the cheapest, highly available, and natural occurring-phenolic sources, but with reduced bioavailability. The main reason for their low bioavailability stems from their structural position being esterified or covalently bound to arabinoxylan; only free and some conjugated phenolic are accessible and absorbed in the small intestine [10]. In addition, this insoluble bound form influences the microbial degradation of arabinoxylan in the human colon [22]; the antioxidant capacity of phenolics is reduced since free hydroxyl groups in the phenolic structure are responsible for the stabilization of free radicals [23]. An in vivo human study by Kern et al. [24] sustains the low bioavailability of the bound form, showing that less than 3% of the ingested WB phenolics were absorbed after 24 h.

Considering these limitations, a priority strategy targets the release of bound phenolic acids in matrices before consumption, for an increased health effect due to the improved bioavailability of compounds. In the literature, several physical pretreatments were used to increase bio-accessibility; they include debranning, dry bran-fractionation, milling, etc. [25,26]. However, bioprocessing via solid-state fermentation (SSF) is gaining popularity due to its positive economic and environmental impacts [27]. The process of fermentation by microorganisms has been used for centuries to increase the nutritional character of foods, and many studies employ different wastes as substrates for the growth of microorganisms [28,29,30]. The enzymes produced by microorganisms under SSF conditions can be used to enhance the release of phenolics from cereal matrices. These enzymes are able to cut bran phenolics’ bonds, increasing their bio-accessibility and biological activity. According to Bhanja et al. [5], SSF is superior to submerged fermentation due to higher productivity, lower water, and energy requirement, easy aeration, lower demand of sterility, resemblance to the natural habitat of microorganisms, easier downstream processing, utilization of cheaper agro-industrial residues as solid substrates, and environmental friendliness. Studies have revealed the effectiveness of SSF in releasing phenolics from different matrices such as rye bran [31], OB [19], soybean [32], buckwheat, wheat germ, and barley [33]. 

Among possible microorganisms used for SSF, *Saccharomyces cerevisiae* (commercially known as baker’s yeast) has high potential, given its GRAS (generally recognized as safe) status for food products, and being a cheap and highly available source. Therefore, *S. cerevisiae* is an economically attractive solution for industrial reproducibility potential of scientific studies, with a major positive impact on agro-industrial waste management via its recirculation. *S. cerevisiae* was found to produce enzymes like β-glucosidases, carboxylesterases, and possibly feruloyl esterases [34,35,36].

The release of phenolics from the matrix through fermentation via extracellular enzymatic action coupled with ultrasound-assisted extraction represents green technology with high efficiency and low costs. According to the most recent comprehensive review study on this topic [27], there are no scientific articles investigating the phenolic profile of oat bran as well its antioxidant activities during solid-state yeast fermentation (SSYF); most studies focus on folate synthesis or folic acid fortification, [37] or using fungi [19]. Regarding WB, more fungi than yeast (much lower for baker’s yeast) were employed in investigating phenolic profiles and antioxidant activities. In existing studies, the SSF conditions used a mix of yeast and synthetic enzymes [38], yeast and lactic acid bacteria [39], or fungi [4,40,41]. Their aim varied among investigations of protein and dietary fiber changes [42,43,44,45], folate production [46], and dephytinization [47]. To the best of our knowledge, only one study used baker’s yeast to investigate the effect of fermentation time and dose [48]; however, it was only for a short period of time (precisely 48 h), while in our study, fermentation time was set at six days. In addition, there is inadequate study on the antioxidant potential of SSYF oat bran and wheat bran, as compared to unfermented oat bran and wheat bran, and whole-grain cereals; comparisons using solid-state fungi fermentations are also insufficient. Therefore, this study aimed to build and evaluate an attractive biotechnological system using *S. cerevisiae* (baker’s yeast) as a GRAS and cheap microorganism under SSF conditions, with the final goal of improving the phenolic content and composition, and antioxidant capacity of industrially derived WB and OB waste, by considering the effect of fermentation time. 

## 2. Materials and Methods

### 2.1. Chemicals

The following chemicals were purchased from Sigma-Aldrich (Steinheim, Germany): 1,1-diphenyl-2-picrylhydrazyl (DPPH), Trolox (TE), Folin-Ciocalteu’s phenol reagent, sodium carbonate, sodium nitrate, ammonium nitrate, hydrochloric acid, aluminum chloride, sodium hydroxide, glucose, acetic acid, acetonitrile, methanol, gallic acid, quercetin, chlorogenic acid, rutin, and cyanidin chloride.

### 2.2. Microorganism and Substrates

A commercially available baker’s yeast preparation (active-dry baker’s yeast—*S. cerevisiae*; Pakmaya^®^, Izmir, Turkey) was used for SSF. Initially, control check for the presence of possible lactic acid bacteria (LAB) in baker’s yeast was assessed. The method involved cultivation in selective cultural media, namely MRS agar, for 48 h at 37 °C (counting plate method). The active-dry yeast was reactivated in 10 mL YPD broth (1% yeast extract, 2% peptone, 2% glucose) and incubated at 30 °C for 24 h. Then, cell density was estimated by spectrophotometry at 600 nm. Cell suspension was adjusted with a medium up to 10^7^ colony-forming units/g [49]. The commercially available white WB and OB (Solaris^®^, Bucharest, Romania) were used as substrates for cell growth and the degradation pattern of polysaccharide-phenolic acid complexes was investigated with time.

### 2.3. Solid-State Yeast Fermentation (SSYF)

The WB and OB samples were milled using an analytical mill (A 10 IKA, Sartorom, Bucharest, Romania), sieved through a 40-mesh screen, and sterilized at 121 °C for 15 min [48]. The SSF culture was performed in 500-mL Erlenmeyer flasks containing 100 g of sterile material, moisturized up to a humidity of 70% [4] (*w/w*) in case of WB, and 45% (*w/w*) in case of OB [19]. The flasks were inoculated aseptically with 5 mL of yeast suspensions (10^7^ CFU/mL) per 100 g of dry weight (DW), mixed properly and incubated for 6 days at 30 °C under static conditions. The WB and OB samples treated with the same procedure and inoculated with an equal amount of sterile water (5 mL) were used as controls. Incubation for each type of substrate was carried out in triplicate. To determine phenolic content and composition, antioxidant activity and cell viability of the samples, aliquots (~10 g) from the Erlenmeyer flasks were removed every day (intervals of 24 h), thermally inactivated, and stored at −20 °C until further analysis.

### 2.4. Cell Viability

Yeast cell viability was determined by diluting 1 g of each fermented WB and OB sample taken from the Erlenmeyer flasks in 9 mL of sterile saline solution (0.8% NaCl). One hundred µL of different dilutions were inoculated on YPD agar plates and incubated for 24 h at 30 °C. The viability of *S. cerevisiae* cells was assessed by plate counting and expressed as logarithmic values of the colony-forming units per mL of the sample (log10 CFU/mL) [50,51]. Microscopic examination was used to investigate viable yeast cells, and as a second control check for other microorganisms. For this, a loop of inoculated fermentation media was put on a glass laboratory lamella, dyed with methylene blue, and examined at 400× magnification [52].

### 2.5. Ultrasound-Assisted Extraction (UAE)

Extractions of the samples were carried out according to a previously reported method [3,53], with slight modifications. The fermented mass (10 g) taken from the Erlenmeyer flasks was dried in an oven (VENTI-Line 112 Prime, VWR, Darmstadt, Germany) at 60 °C for 24 h. The dried substrates (fermented and control) were ground in an electric grinder and transferred to an Erlenmeyer flask and defatted with hexane (ratio 1:5 *w/v*, 5 min, three times) at room temperature. Defatted samples were air-dried at room temperature for 24 h, and then ultrasonically extracted (Elmasonic E15H, Elma, Singen, Germany) thrice with 80% methanol at a 1:5 ratio (*w/v*) at 40 °C for 1 h. After centrifugation at 2.000× *g* for 10 min, the supernatant was filtered using Whatman No. 1 filter paper, and the combined supernatant was concentrated to dryness with a rotary evaporator at 40 °C. The dried extracts were reconstituted in methanol (2 mL), filtered through a 0.45 μm syringe filter, and subsequently subjected to analysis (total phenolic content [TPC] assay, HPLC analysis of phenolics composition, DPPH radical-scavenging activity). All analyses were performed in triplicates.

### 2.6. Determination of TPC

TPC was determined according to the Folin-Ciocalteu method [54,55,56]. Briefly, each extract (25 µL) was mixed with 125 µL of Folin-Ciocalteu reagent (0.2 N). After 2 min of incubation at room temperature, 100 µL of 7.5% (*w/v*) sodium carbonate solution (Na_2_CO_3_) was added to the mixtures. They were then allowed to stand at room temperature for 2 h in the dark and absorbance was measured at 760 nm. TPC was expressed as gallic acid equivalents (GAE) in mg/g DW of bran from the calibration curve of a gallic acid standard solution (0.01–1 mg/mL). All samples were performed in triplicate.

### 2.7. High-Performance Liquid Chromatography (HPLC-DAD-ESI-MS) Analysis of Phenolic Compounds

We used an HPLC system consisting of an Agilent 1200 HPLC with DAD detector, coupled with an MS-detector single-quadrupole Agilent 6110. Separation was performed at 25 °C on an Eclipse column, XDB C18 (4.6 × 150 mm, 5 µm) (Agilent Technologies, Santa Clara, CA, USA). Two solvents were used for the mobile phase: 0.1% acetic acid in distilled water (*v/v*) (solvent A) and 0.1% acetic acid in acetonitrile (*v/v*) (solvent B). The gradient elution (expressed in% B) used was: 0 min, 5% B; 0–2 min, 5% B; 2–18 min, 5–40% B; 18–20 min, 40–90% B; 20–24 min; 90% B; 24–25 min, 90–5% B; 25–30 min, 5% B. 

Each type of extract (20 μL) was injected into the column. The flow rate was 0.5 mL/min, and detection was performed at 280 nm and 340 nm. Components were identified by a comparison of their retention times, UV visibility, and mass spectra with those in the literature under identical analysis conditions. For MS fragmentation, the ESI (+) module was applied, with a scanning range between 100 *m/z* and 1200 *m/z*, capillary voltage of 3000 V, at 300 °C and with nitrogen flow of 7 L/min. The eluent was monitored by DAD, and the absorbance spectra (200–600 nm) were collected continuously during the course of each run [54]. All the samples were injected in triplicate. Data analysis was performed using Agilent ChemStation Software (Rev B.04.02 SP1, Palo Alto, CA, USA).

### 2.8. DPPH Free-Radical-Scavenging Assay

The free radical scavenging activity of the UAE methanolic extracts was measured in terms of proton-donating ability. The reaction between DPPH and antioxidants in the extracts was monitored using a BIOTEK spectrometer at 515 nm. The stock solution of DPPH was freshly prepared by dissolving 80 μM DPPH in 98% methanol, sonicated for 15 min, and stored in the dark at room temperature. The methanol solution was used as a blank, then 2.8 mL of the DPPH solution and 400 μL of the sample were used for each assay; absorbance was recorded after 30 min, taking into consideration the fact that the DPPH solution decolorizes from purple to yellow in the presence of a proton donor. The calibration curve was performed with Trolox, using various dilutions (500 μM, 250 μM, 125 μM, down to 3.95 μM), and then absorbance was recorded for all samples. The antioxidant activity of the extracts evaluated—the equivalent factor F (mM Trolox)—was reported at 100 g DW. The percentage of inhibition (I%) was calculated as follows:

[1 − (test sample absorbance/blank sample absorbance)] × 100(1)

### 2.9. Statistical Analyses

All tests were conducted in triplicate and the results were expressed as the means ± standard deviation (SD). Statistical differences among samples were estimated using one-way ANOVA (Tukey multiple comparison tests) using GraphPad Prism Version 8.0.1 (Graph Pad Software Inc., San Diego, CA, USA). Differences between means at the 5% level were considered statistically significant. Correlations between antioxidant capacity and phenolic content were determined using Pearson’s correlation.

## 3. Results and Discussions

### 3.1. Cell Viability

**Figure 1:**
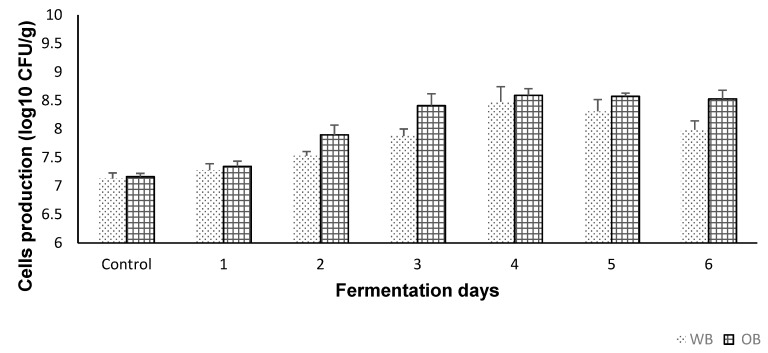
Cell viability (plate counting method) of the extracts (control and fermented (6 days)). Values are expressed as mean values ± SD, log10 CFU/g, *n* = 3, GraphPad Prism Version 8.0.1, Graph Pad Software, Inc. (San Diego, CA, USA).

The control test via selective cultural media for LAB identification showed no growth. Moreover, the microscopic examination showed no other microorganisms present. The cell growth profile of *S. cerevisiae* during WB and OB fermentation is shown in Figure 1. Slow growth was observed on the first day of fermentation for both substrates. Starting with day 2, population growth gradually increased, *S. cerevisiae* reaching a maximum viable cell number of 8.47 ± 0.26 log CFU/g for WB, and 8.58 log ± 0.11 log CFU/g for OB, respectively, on day 4 of fermentation. On days 5 and 6, a small decrease in cell production was observed, which may be attributed to a decline in nutrient source, and accumulation and/or toxicity of waste products. Overall, both cereal brans were considered good substrates for the growth of *S. cerevisiae* cells.

### 3.2. Total Phenolic Content

**Figure 2:**
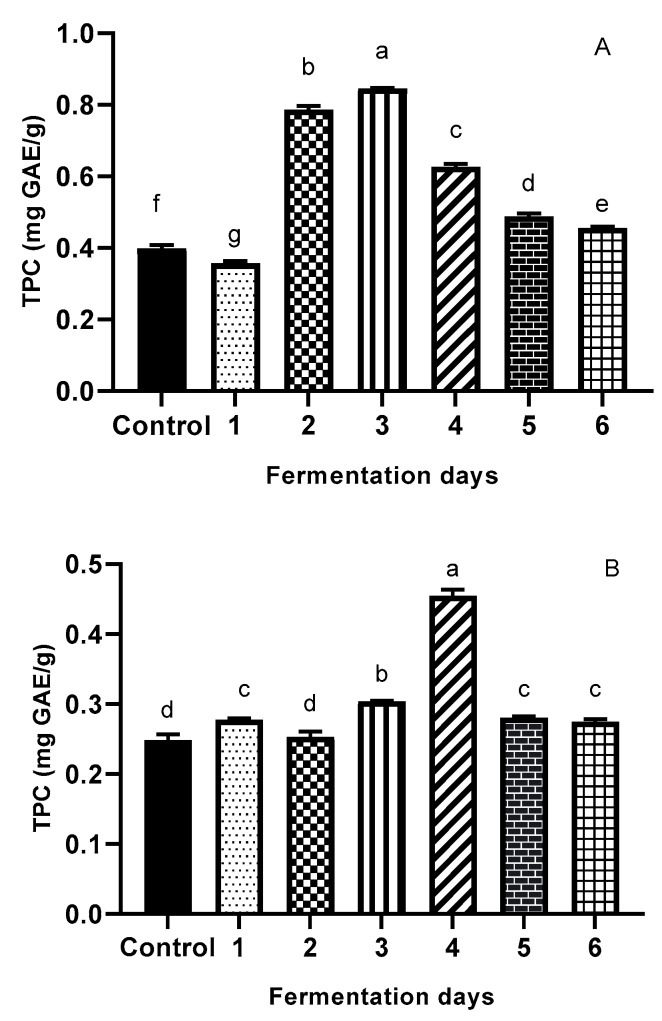
Total phenolic content (Folin–Ciocalteu method) (**A**) illustrating WB; (**B**) illustrating OB of the extracts (control and fermented (6 days)). Total phenolic content of the extracts is expressed as gallic acid equivalents (GAE) in mg/g sample of waste. Values are expressed as mean values ± SD, μg/g, *n* = 3, and are followed by different letters (a–f), indicating significant differences (*p* < 0.05) between days of fermentation (One-way ANOVA - Multiple comparison test - Tukey multiple range test (*p* = 0.05); GraphPad Prism Version 8.0.1, Graph Pad Software, Inc., San Diego, CA, USA).

The TPC of extracts measured by the Folin-Ciocalteu method is shown in Figure 2A,B for both cereal brans. An 80% aqueous methanol represents the most suitable solvent for total phenolic acids extraction, given the highest yield of extractable WB and OB, as previously reported in the literature [3,4,53]. There were significant differences (*p* < 0.05) between the TPCs of the control (time 0) and fermented bran materials, as well as between fermentation days. As shown in Figure 2A,B, maximum TPC was registered on day 3 of fermentation for WB (0.84 ± 0.05 mg GAE/g DW), and day 4 for OB (0.45 ± 0.02 mg GAE/g DW). As an overall result, it can be stated that fermented samples of WB and OB had higher phenolic contents than their non-fermented counterparts. This is mainly due to increases in methanol-extractable phenolic compounds and can be attributed to the possible enzymes produced, enhancing the availability of phenolic compounds. In the case of WB, a 112% increase in TPC value was observed on day 3 of fermentation; for OB, an 83% increase in TPC value was observed on day 4, compared to the control. Fermentation-induced structural breakdown of cereal cell walls may occur, leading to the liberation of various bioactive compounds [31].

Other authors also reported an increase in TPC after fermentation. In a study by Moore et al. [48], increases of 50%, 100%, and 69% in fermented WB versus controls were reported for three commercially available yeast preparations. Cai et al. [19] reported a dramatically increase in TPC and flavonoids of oat subfractions after SSF with filamentous fungi. Dulf et al. [57] showed that total phenolic levels in apricot pomace (*Prunus armeniaca* L.) increased by more than 70% after fermentation with *Rhizopus oligosporus* and by more than 30% for fermentation with *Aspergillus niger.* Lee, Hung, and Chou [58] found that the β-glucosidase enzyme produced by microorganisms during SSF was responsible for the phenolic content increase in black bean koji. Because many OB phenolic compounds are esterified and insoluble-bound [59], the increase of TPC in fermented OB might be attributed to the effect of certain enzymes (depending on the microorganisms used), such as glycoside hydrolase, cellulose, esterases, β-glucosidases, which could potentially release bound phenols during fermentation in a time-dependent manner. From Figure 2A,B, the data clearly indicate that the increased TPC ratios were dependent on fermentation time.

The increase in TPC was observed until day 3 of fermentation (72 h) for WB and day 4 of fermentation (96 h) for OB; thereafter, TPC decreased. Bhanja et al. [60] reported TPC of fermented wheat in the range between 30.6 and 158.1 mmol GAE/g. Schmidt and Furlong [61] reported that the changes produced by fermentation in phenolic acids profiles depend on the type of substrate, the microorganism used, and the conditions of fermentation.

### 3.3. Antioxidant Activity

**Figure 3:**
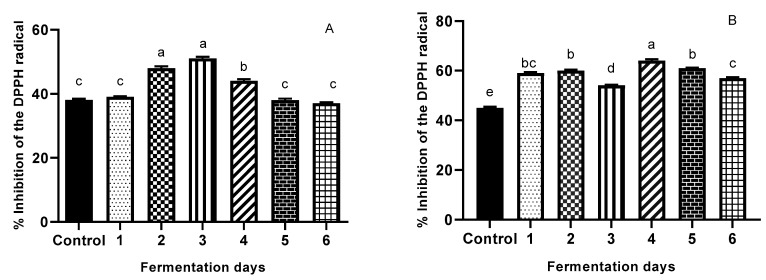
Antioxidant activity (DPPH free-radical-scavenging assay) (**A**) illustrating WB; (**B**) illustrating OB of the extracts (control and fermented (6 days)). The percentage inhibition (I%) was calculated as [1 − (test sample absorbance/blank sample absorbance)] × 100. Values are expressed as mean values ± SD, I%, *n* = 3, and are followed by different letters (a–f) indicating significant differences (*p* < 0.05) between days of fermentation (One-way ANOVA - Multiple comparison test -Tukey multiple range test (*p* = 0.05)-GraphPad Prism Version 8.0.1, Graph Pad Software, Inc., San Diego, CA, USA).

Changes in the antioxidant activity of the extracts were assessed by measuring the DPPH radical-inhibition capacity (RIC); the results are presented in Figure 3A,B. During yeast SSF, statistically significant increases (34.21%) (*p* < 0.05) in RIC of WB waste was registered on day 3. The OB extract exhibits the highest inhibition percentage on day 4 of fermentation with a relative percentage increase of 42.22%. The increase in antioxidant activities of both SSYF cereal wastes shows that antioxidant activity depends on the presence of all polyphenols that have increased during the fermentation treatment, showing, in general, a maximum on day 3 for WB, and day 4 for OB, respectively. The increased antioxidant activity may be caused by *S. cerevisiae* fermentation-induced changes in TPC. These observations agree with the results of Zhang et al. [4], who found that bioprocessing (6 days) by different strains enhances the bioactive content and composition value of WB by increasing the total antioxidant activity. Moore et al. [48] showed that fermented WB (48 h) by commercial yeast was able to significantly increase antioxidant properties owing to its ability to remove TPC radicals from 13 to 19, and DPPH radicals from 50% to 100%. Xiao et al. [62] found that SSF (8 days) significantly (*p  *< 0.05) enhanced the content of phenolics, avenanthramides, and flavonoids; and antioxidant activities of oats. Compared to WB, OB presented a stronger antioxidant activity, a fact that could be explained by the presence of phenolic acids like avenanthramides, which are well known for their antioxidant effect, as well as *p*-coumaric and sinapic acids.

### 3.4. Fermentation Time

Fermentation time is a kinetic parameter with relevant importance for optimal enzyme production. In general, enzyme production is directly dependent on fermentation time until it reaches a maximum, thereafter declining in production and activity. This decrease may be explained by a decline in nutrient availability, accumulation and/toxicity of waste products, and a decrease in enzyme stability [63]. For example, the maximum β-glucosidase activity in white grape juice by several *S.cerevisiae* strains and non-*Saccharomyces* yeast strains, was reached in the first 24–48 h of fermentation [64]. Optimal β-glucosidase production by the yeast *Debaryomyces pseudopolymorphus* was obtained over 72 h using a nutrient medium containing cellobiose [65]. About 10 different strains of *S. cerevisiae* were screened for β-glucosidase production in Thai fruits and fruit-derived beverages, concluding that the 24 h incubation achieved the highest yield [49]. The esterase synthesis from *S. cerevisiae* on sub-merged fermentation over 35 h was studied, suggesting that an optimal glucose medium may provide the highest production [66]. Highest cellulase production from *S.cerevisiae* on pineapple peel substrate was achieved on day 3 [67]. In addition, the endoglucanase synthesis from *Streptomyces diastaticus* was investigated using a mix of sugarcane bagasse, oat bran, and corn steep liquor, reporting the highest level after day 5, and a good level on day 4 [68].

#### Effect of Fermentation Time on Antioxidant Properties of WB and OB

The results for both TPC and DPPH (Figure 2A,B and Figure 3A,B) illustrate that the antioxidant properties of SSYF wheat and oat bran were dependent on SSF time. In the case of WB, data for DPPH (Figure 3A) showed no significant increase during day 1, followed by a significant increase in DPPH on day 2 (26.32%), reaching maximum increase on day 3 (34.21%); the increase was not statistically significant between day 2 and day 3. These results are in agreement with previous findings by Sandhu et al. [69], who found that TPC and metal chelating activities of several wheat cultivars increased to the maximum until day 4 of fermentation. These results may be explained by the fact that in the first two days, yeast cells could be producing enzymes capable of hydrolyzing bound antioxidative compounds, being the most proliferative due to a high rate of nutrient availability; while after day 3, a possible drop in nutrient availability may occur. Results for TPC (Figure 2A) show a similar trend with the highest percentage increase versus a control occurring on day 3 (112%), but with an initial significant decrease in TPC during day 1 (−10.48%). The reduced TPC can be explained by the fact that the presence of yeast contributed to simple phenolic conversion and the depolymerization of high molecular weight phenolic compounds in WB. In addition, the reduced TPC does not necessarily imply a reduction in antioxidant activity of WB, because yeast might deplete the available glucose molecule bound to the phenolic compounds, resulting in the production of free aglycones with a higher number of hydroxyl groups [70].

**Figure 4:**
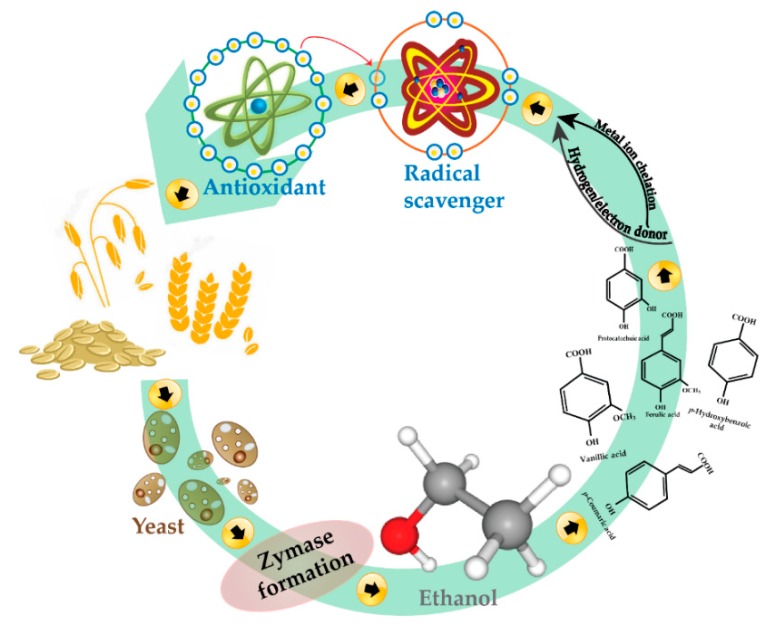
A schematic showing how the effect of yeast fermentation on the antioxidant activity of cereals takes place.

In the case of OB, data for DPPH (Figure 3B) show a non-linear increase in the first three days of fermentation; maximum increase in antioxidant activity was registered (42.22%) on day 4. On day 1, there was a significant increase in antioxidant activity (31.11%), followed by a plateau on day 2, and a significant drop on day 3 (−10%). This decrease could be attributed to a lack of nutrient availability, which in turn might direct the bioprocess for glucose depletion specific enzymes. This finding is in agreement with Cai et al. [71], who concluded that the antioxidant activities of fermented oats significantly increased after three days of fermentation (*p*  <  0.05). Another study [19] reported an increase in antioxidant activities of fungi-fermented oat after three days; it must be mentioned that fermentation time is also microorganism-dependent. Considering our TPC results (Figure 2B), a similar trend is observed, with the highest increase (83% vs. control) on day 4, followed by day 3 (22.24% increase vs. control), both significantly different from the other days of fermentation (*p* < 0.05). Figure 4 illustrates how the effect of yeast fermentation on antioxidant activities of WB and OB takes place. In summary, increased antioxidative activity in fermented cereal bran might be due to an increase in the release of antioxidant compounds via microbial hydrolysis. Yeast fermentation induces the structural breakdown of cell walls, therefore leading to the liberation of various antioxidant compounds. This mechanism also involves exposure of microorganisms to oxidative stress during fermentation; therefore, the cells may evolve protective mechanisms involving enzymatic antioxidation, which may contribute to the antioxidative effect of fermentation.

### 3.5. Analysis of Phenolic Compounds by HPLC-DAD-ESI-MS

The ultrasound-assisted extraction of phenolic compounds was improved upon by SSYF, which helped in the release of bound compounds, like ferulic acid, apigenin glucoside (WB substrate), and avenanthramides (OB substrate). The analyzed samples contained 11 phenolic compounds that belong to three phenolic groups: hydroxybenzoic acids, hydroxycinnamic acids, and flavones. From the hydroxybenzoic group, protocatechuic acid, vanillic acid, and dihydroxybenzoic acids were identified. Apigenin-glucoside was the only flavone identified from WB. The seven hydroxycinnamic acids detected were: ferulic acid, caffeic acid, *p*-coumaric acid, sinapic acid, avenanthramide 2c, avenanthramide 2p, and avenanthramide 2f (Table 1); avenanthramides were reported as unique-oat compounds.

#### 3.5.1. Changes in Phenolic Composition During Fermentation

The changes in methanolic soluble phenolic compounds of WB and OB are presented in Table 2; Table 3, respectively, expressed as μg GAE/g DW. The changes in all phenolic acids content were assessed during fermentation.

The phenolics composition of WB (Table 2) was improved most (highest relative percentage increase) on day 3 (ferulic acid +56.6%, vanillic acid +259.3%, dihydroxybenzoic acid +161.2%, apigenin-glucoside +15.2%). The major phenolic acid identified was dihydroxybenzoic acids, which significantly increased in time, reaching its maximum value on day 3 (115.8 ± 5.6 μg/g), followed by day 4 as next percentage increase (147.6%). Thereafter, it decreased significantly, but not below the control value. Considering the high concentration found, dihydroxybenzoic acids might also include their isomers, such as 3,4-Dihydroxybenzoic acid (Protocatechuic acid), and *p*-hydroxybenzoic acid, as recently reported in WB [4,72]. Ferulic acid was amongst the major phenolic compound identified. Ferulic acid started to increase significantly from day 1 of fermentation, reaching the maximum value (34.3 ± 0.2 μg/g) on day 3; it then decreased. Its decrease may be explained considering its possible bioconversion by yeast into vanillin. The first step involving the transformation of ferulic acid to vanillyl alcohol might be done by *S. cerevisiae,* which transfers ferulic acid to vanillic acid by propenoic chain degradation [73], subsequently resulting in oxidative decarboxylating to 2-methoxyhydroquinone. With regard to vanillic acid, a significant increase starting with day 2 of fermentation was observed, and therefore, the aforementioned mechanism might be relevant.

Apigenin-glucoside, the only flavone compound found in WB, had the highest increase on day 3. According to Radenkovs et al. [74], enzymatic action is most efficient in releasing linked flavonoid forms in WB. The high antioxidant activity of WB can also be explained by the presence of this compound, previously reported with significant in vitro antioxidant activity [75,76,77].

All the phenolic acids identified in the control and fermented OB during the six days of fermentation are reported in Table 3. OB extracts showed a higher variety of phenolic acids, which increased significantly on day 4. Dihydroxybenzoic acids were, as well, the major compound identified with the highest concentration (52.15 ± 1.07 μg/g) on day 4, followed by avenanthramides (2c, 2p, 2f). Avenanthramide 2f had the highest increase (48.45% vs control), followed by ferulic acid (21.24%). The other phenolic acids identified were protocatechuic, caffeic, and vanillic acids with the highest percentage increases of +42.87%, +25.51%, and +15.13%, respectively, registered on day 1. *p*-coumaric acid content increased significantly every 24 h, reaching the highest value (3.68 ± 0.01 μg/g) on day 4. Compared to the control, sinapic acid reached maximum increase (+57.07%) on day 2, followed by day 3. Several studies have identified phenolic compounds in oat, precisely caffeic and ferulic acids as main phenolic acids [59]; vanillic, sinapic, *p*-coumaric, and protocatechuic acid have also been reported [19,78].

The reported hydroxybenzoic acids in oats [79] include protocatechuic, syringic, vanillic, *p*-hydroxybenzoic and gallic acids, while hydroxycinnamic acids in oats are ferulic, *p*-coumaric, caffeic, and sinapic acids, which were mostly in line with our findings. The structure of avenanthramides comprises of an amide conjugate of anthranilic acid and hydroxycinnamic acids. The three identified ones are esters of 5-hydroxyanthranilic acid with *p*-coumaric (2p aka A), ferulic (2f aka B), and caffeic (2c aka C) acids, consistently found in OB [80,81].

The content of oat phenolics reported in other studies are mostly in line with our findings, but there are a few differences. Ferulic acid was reported to be the major component in several studies [79,82,83]. *p*-coumaric acid was previously found to be in higher quantities, in agreement with Soycan et al. [84]. Avenanthramide-2f was the predominant avenanthramide, which was in line with findings by Chen et al. [85] and Soycan et al. [84]. However, the concentrations of avenanthramide-C reported here are lower than in other studies [79,83]. These variations may be due to differences in samples used (e.g., our study analyzed the OB), the methods used for extraction and analysis, and genetic and environmental factors.

#### 3.5.2. Possible Implications of SSF in the Increased Phenolic Acids Content

Molecular weight, degree of polymerization, and microstructure are extremely important in analyzing the extractability percentage and, therefore, antioxidant ability of phenolic compounds [74]. The antioxidant activity of control WB and OB extracts are in line with previous findings [19,48,84,86]. In accordance with other SSF studies on WB [4], black bean [58], and rice bran [23], the bioprocess delivered a significant increase of TPC in comparison with the control, together with their antioxidative activity. The naturally bound form of phenolics may have a certain role in the reduced availability of free hydroxyl groups needed for free-radical scavenging ability. Therefore, the action of the enzyme, followed by a further release of functional groups (OH) of polyphenol derivatives, may explain the correlation between higher phenolic content and higher antioxidative capacity in the fermentation process. However, the influence of each phenolic acid and their contribution to the total antioxidant capacity needs to be further investigated. According to the literature, the presence of the CH=CH–COOH group is the main reason for the higher antioxidative efficiency of the hydroxycinnamic acids versus the hydroxybenzoic acids [3]. When compared to the methoxy group at the C3, the hydroxyl group was reported to have a higher impact on the radical-scavenging activity [87,88]. The number of OH groups, their position (ortho groups stabilize each other via electron delocalization), and methylation are all contributing factors to antioxidant activity strength. For example, ferulic and caffeic acids are more efficient than benzoic and vanillic acids. Considering this, the correlation between higher TPC and higher antioxidant activity might be explained, as well. It is very clear that days of fermentation have a strong influence on antioxidant activity enhancement, considering cell growth, nutrient availability and accumulation and/toxicity of waste products, and a decrease in the stability of the enzyme itself. 

**Figure 5:**
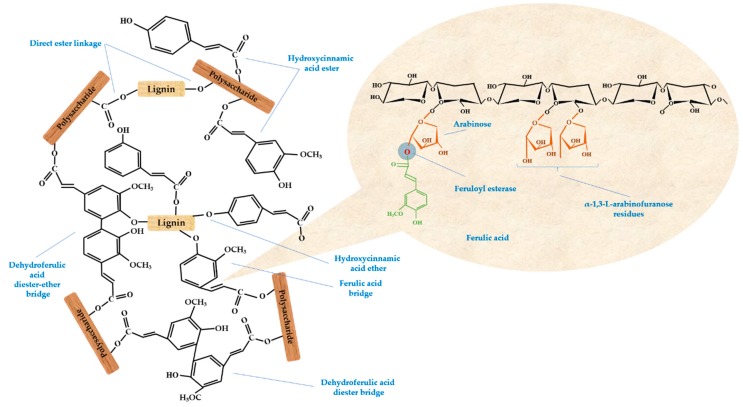
Reported linkages between dietary fiber components and phenolic acids with a loop on ferulic acid structure esterified to arabinoxylan and possible microorganisms-produced enzymes for increased bioavailability.

Our approach of yeast treatment increased the phenolic content, which in turn resulted in the solubilization of phenolics-linked cell wall carbohydrates. Therefore, the digestibility, accessibility, and bioavailability of the cereal substrate were probably improved. Figure 5 illustrates the possible linkages between dietary fibers and phenolic acids presents in cereal grains, with a loop on the ferulic acid structure. As ferulic acid is consistently reported as a major antioxidant [10], its linkages are important to be cleaved for increased intestinal absorption. In wheat, ferulic acid is bound to arabinoxylans and therefore has low bioavailability, while only the free form is easily absorbed, as it is bioaccessible. Zhao et al. [89] showed how different molecular sizes of ferulic sugar esters are differently absorbed within the intestinal membrane. Moreover, Anson et al. [90] reported that yeast treatment with *S. cerevisiae* may increase the bioaccessibility of ferulic acid in wheat. Our tested microorganism was a GRAS strain (*S. cerevisiae*), generally considered a food-grade species and commonly applied in the fermentation industry for the production of microbial-derived food ingredients [3,91,92,93]. The present findings could showcase SSF as a promising method to achieve higher bioavailability of phenolic acids naturally present in cereal bran, and to possibly improve health benefits in humans.

The results of this paper represent a step forward in the yeast-based enrichment of oat bran and wheat bran, considering the use of baker’s yeast for antioxidant delivery in the human diet. Of major importance are the setpoints of fermentation time, considering the release pattern of phenolic compounds based on significant differences (*p* < 0.05) between each day of fermentation.

### 3.6. Pearson’s Correlation

Pearson’s correlation coefficient (*r*) was obtained after comparing the antioxidant capacity values with the total phenolic content. The r values above 0.9 are considered to show a correlation. For WB and OB, Pearson’s correlation showed a high interrelationship between the phenolic content and the DPPH antioxidant method (*r* = 0.95 [WB], *r* = 0.90 [OB]). The positive correlations between the DPPH antioxidant assay and the phenolic content suggest that phenolic compounds present in ultrasound-assisted methanolic extracts have a high contribution to the antioxidant activity of both brans, native and fermented, respectively. Bhanja et al. [60] found a high positive correlation between TPC and DPPH radical scavenging activity for wheat fractions fermented with *A. awamori* (*r* = 0.977, *p* < 0.05), and Sandhu et al. [69] highly positively correlated TPC and DPPH of wheat cultivars fermented with *A. awamorinakazawa* (*r* = 0.984, *p* < 0.05).

## 4. Conclusions

The results revealed that commercially available baker’s yeast used for SSF was effective in increasing the TPC, DPPH radical activity, and phenolic composition of WB and OB, while demonstrating the huge significance of fermentation time. The one-way ANOVA-Tukey test was used to study the effect of fermentation time by using the multiple comparison approach. The optimal fermentation duration in terms of TPC content and antioxidant activity was three days for WB and four days for OB. The increase in antioxidant activity significantly correlated with TPC. Therefore, SSYF can be successfully applied to enriching matrices in phenolic acids and to increase their antioxidant activity; however, the results should be closely monitored with regard to fermentation time.

Also considering the low cost and high availability of substrates (commercial cereal bran) and baker’s yeast used (commercially active-dry yeast), the wet and dry milling industries may better valorize their wastes by a possible re-introduction in technological flow via the design of functional food products or functional ingredients with low production costs. This study may also promote a sustainable approach to feed the increasing world population (10 billion people by 2050) by promoting recirculation of resources in the context of a circular economy.

## Figures and Tables

**Table 1:** Identification of phenolic compounds in fermented samples on WB and OB substrates.

Substrate	Peak No. WB Substrate	Retention Time Rt (min)	[M+H]^+^ (m/z)	UV λmax (nm)	Compound	Class/Subclass
WB Substrate	1	2.94	156	250	Dihydroxybenzoic acids	Hydroxybenzoic acid
	2	13.89	169	255, 300	Vanillic acid	
	3	14.31	433	272, 340	Apigenin-glucoside	Flavone
	4	17.12	195	322	Ferulic acid	Hydroxycinnamic acid
OB Substrate	1	2.94	156	250	Dihydroxybenzoic acids	Hydroxybenzoic acid
	2	6.05	156	260, 280	Protocatechuic acid	
	3	13.51	181, 163	320	Caffeic acid	Hydroxycinnamic acid
	4	13.89	169	255, 300	Vanillic acid	Hydroxybenzoic acid
	5	15.78	165	319	*p*-Coumaric acid	Hydroxycinnamic acid
	6	16.85	225	321	Sinapic acid	
	7	17.12	195	322	Ferulic acid	
	8	17.97	316	330	Avenanthramide 2c	
	9	19.81	300	330	Avenanthramide 2p	
	10	20.37	330	330	Avenanthramide 2f	

**Table 2:** Phenolic compounds analysis of the fermented wheat bran samples/fermentation time.

Fermentation Days	di-OH Benzoic ac	Vanillic ac	Apigenin-Glucoside	Ferulic ac
0	44.3 ± 2.1^f^	5.9 ± 0.2^c^	10.7 ± 0.6^c^	21.8 ± 0.1^d^
1	74.8 ± 1.7^e^	5.6 ± 0.2^c^	9.1 ± 0.2^d^	21.9 ± 0.1^d^
2	88.7 ± 2.6^c^	20.8 ± 1.2^a^	14.6 ± 0.7^b^	33.9 ± 0.3^b^
3	115.8 ± 5.6^a^	21.4 ± 1.2^a^	15.2 ± 0.3^a^	34.2 ± 0.2^a^
4	109.7 ± 4.7^b^	8.5 ± 0.9^b^	14.5 ± 0.3^b^	31.6 ± 0.07^c^
5	78.2 ± 2.8^d^	5.9 ± 0.8^c^	9.8 ± 0.2^d^	20.0 ± 0.2^d^
6	74.3 ± 3.3^e^	5.1 ± 0.7^c^	8.4 ± 0.08^e^	13.3 ± 0.1^e^

Values (expressed as mean values ± SD, μg/g, *n* = 3) in the same column followed by different letters. (a–f) indicate significant differences (*p* < 0.05) between days of fermentation (One-way ANOVA - Multiple comparison test -Tukey multiple range test (*p* = 0.05)- GraphPad Prism Version 8.0.1, Graph Pad Software, Inc., San Diego, CA, USA).

**Table 3:** Phenolic compounds’ analysis as per fermented oat bran samples/fermentation time.

Fermentation Days	di-OH Benzoic	Protocatechuic	Caffeic	Vanillic	*p*-Coumaric	Sinapic	Ferulic	Avenantr. 2c	Avenantr. 2p	Avenantr. 2f
0	38.76 ± 0.38^f^	2.79 ± 0.08b^c^	3.30 ± 0.04^d^	2.01 ± 0.02^b^	2.18 ± 0.01^f^	4.68 ± 0.01^f^	7.80 ± 0.04^e^	8.75 ± 0.04^f^	7.72 ± 0.06^e^	10.19 ± 0.09^e^
1	43.68 ± 0.34^e^	3.98 ± 0.02^a^	4.14 ± 0.05^a^	2.32 ± 0.01^a^	2.73 ± 0.03^d^	5.48 ± 0.02^e^	9.68 ± 0.02^a^	11.09 ± 0.02^d^	9.85 ± 0.04^c^	13.45 ± 0.04^c^
2	47.17 ± 0.41b^c^	3.93 ± 0.05^a^	3.93 ± 0.06^b^	1.99 ± 0.01^b^	3.14 ± 0.03^c^	7.35 ± 0.03^a^	9.24 ± 0.03^c^	11.66 ± 0.05^ab^	10.34 ± 0.08^abc^	14.29 ± 0.09^b^
3	47.31 ± 0.13^b^	2.75 ± 0.05^b^	3.51 ± 0.02^c^	1.62 ± 0.05^cd^	3.36 ± 0.03^b^	6.02 ± 0.02^d^	8.17 ± 0.04^d^	10.26 ± 0.07^e^	8.22 ± 0.02^d^	11.90 ± 0.11^d^
4	52.15 ± 1.07^a^	2.97 ± 0.03^b^	3.86 ± 0.01^b^	1.67 ± 0.04^c^	3.68 ± 0.01^a^	7.09 ± 0.02^b^	9.46 ± 0.03^b^	11.77 ± 0.10^a^	10.97 ± 0.08^a^	15.12 ± 0.02^a^
5	46.29 ± 0.23^c^	2.42 ± 0.06^c^	2.31 ± 0.07^e^	1.67 ± 0.02^cd^	2.62 ± 0.04^e^	6.67 ± 0.02^c^	8.44 ± 0.03^d^	11.56 ± 0.09^b^	10.15 ± 0.04^b^	13.81 ± 0.08^bc^
6	45.79 ± 0.20^d^	2.25 ± 0.05^c^	1.60 ± 0.09^f^	1.52 ± 0.02^d^	1.83 ± 0.03^g^	5.85 ± 0.04^d^	6.57 ± 0.02^f^	11.28 ± 0.07^c^	9.84 ± 0.03^c^	13.57 ± 0.08^c^

Values (expressed as mean values ± SD, μg/g, *n* = 3) in the same column followed by different letters (a–f) indicate significant differences (*p* < 0.05) between days of fermentation (One-way ANOVA - Multiple comparison test -Tukey multiple range test (*p* = 0.05)-GraphPad Prism Version 8.0.1, Graph Pad Software, Inc., San Diego, CA, USA).
